# Protocol for Measuring Drug–Target Engagement in Mouse Colorectal Cancer Organoids Using NanoBRET Assay

**DOI:** 10.21769/BioProtoc.5752

**Published:** 2026-07-20

**Authors:** Hammed A. Badmos, Colin Steele, Ross Cagan

**Affiliations:** School of Cancer Sciences, Wolfson Wohl Cancer Research Centre, University of Glasgow, Glasgow, UK

**Keywords:** 3D organoid model, Colorectal cancer organoids, NanoBRET, Protein–ligand interaction, Small molecules, Lentiviral transduction, Trametinib

## Abstract

Organoids as a drug discovery platform represent an emerging field that continues to refine its tools. NanoBRET (bioluminescence resonance energy transfer) has emerged as a proximity-based and highly sensitive assay to measure protein–protein and protein–ligand interactions. NanoBRET assays were developed and are currently used for 2D cell line experiments. Here, we present the development of the first organoid-compatible Nanoluciferase (Nluc) for 3D model systems. We utilise the Nluc for NanoBRET assays to test drug–target engagement. We describe steps for seeding, transfecting, and replating of mouse colorectal cancer organoids. In addition, we provide detailed procedures for the NanoBRET assay. Various lines of evidence have shown significant difference in drug response between 2D human cell lines and 3D model systems, including patient-derived organoids. Our protocol provides a template for measuring this difference in the context of drug–target engagement.

Key features

• Development of organoid-compatible Nluc vector.

• Lentiviral delivery and antibiotic selection of MEK1-Nluc organoids.

• NanoBRET assay for the real-time measurement of drug–target engagement in organoids.

• Quantitative measurement of competitive drug binding in living 3D organoids.

• Impact of drug combinations on drug–target engagement in organoids.

## Graphical overview


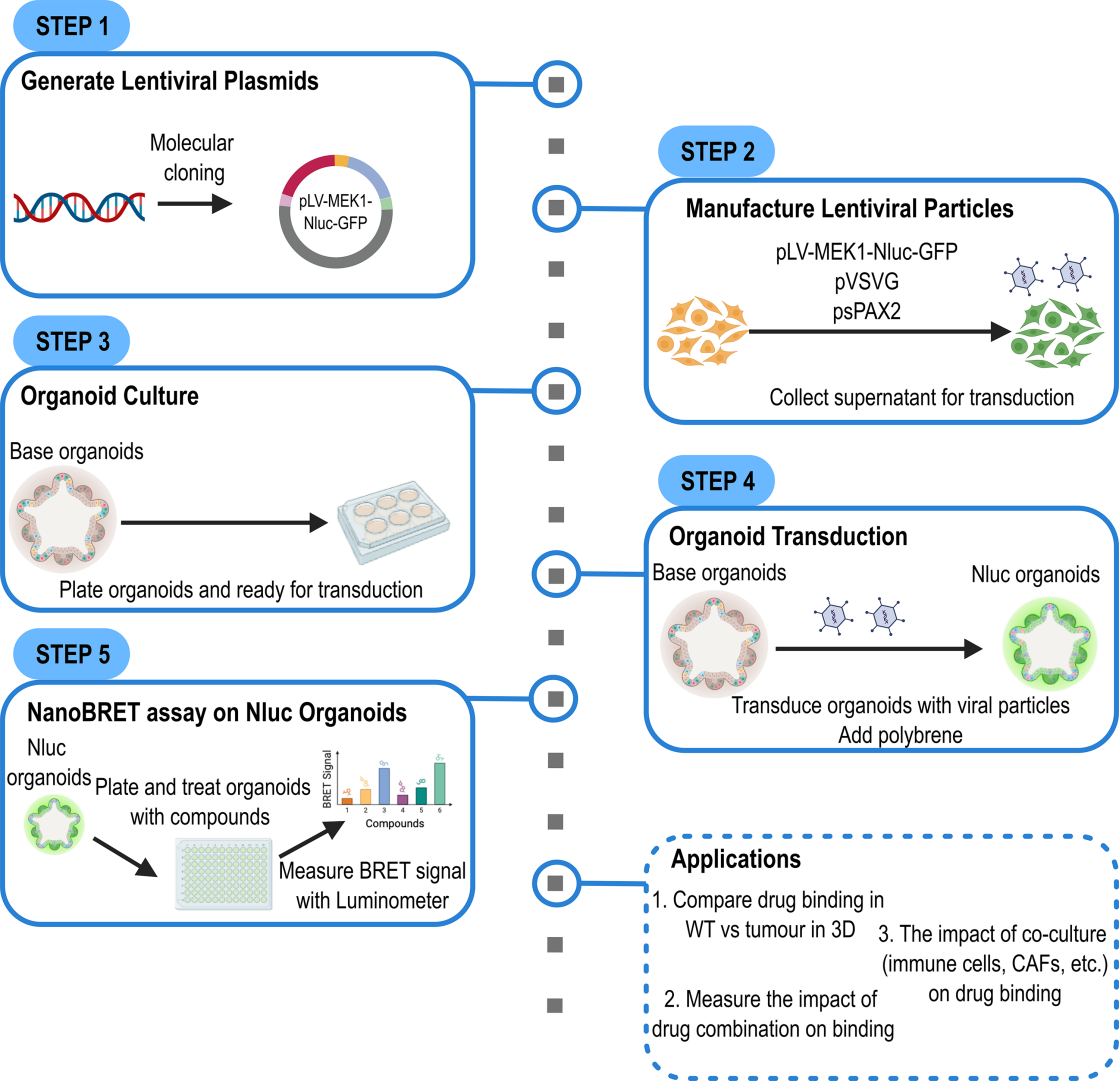


**Schematic overview of the protocol for the development of the NanoBRET system in organoids**

## Background

Organoids have emerged as a powerful tool to study mammalian physiology in normal and disease states [1]. In a three-dimensional (3D) matrix, organoids can self-organise into organotypic structures; for example, intestinal organoids will form crypt and villus structures [2,3]. They provide the convenience of 2D cell culture with a more physiologically relevant 3D structure and are becoming essential research tools across many research areas, including developmental biology, cancer research, and personalised medicine [1,3].

Drug development remains an expensive and challenging process, and organoids are fast becoming a key screening platform. One key problem with targeted therapies is optimising drug–target interactions in vivo [4]. Although 2D cell culture approaches have led to the development of lead compounds and therapeutics, a wide discrepancy is often observed between drug activity in 2D cultures and patients due to the lack of cellular context required for proper in vivo drug–target engagement [4,5]. The complexity and heterogeneity of the organoid system provides one approach to help bridge this gap.

**Figure 1.**
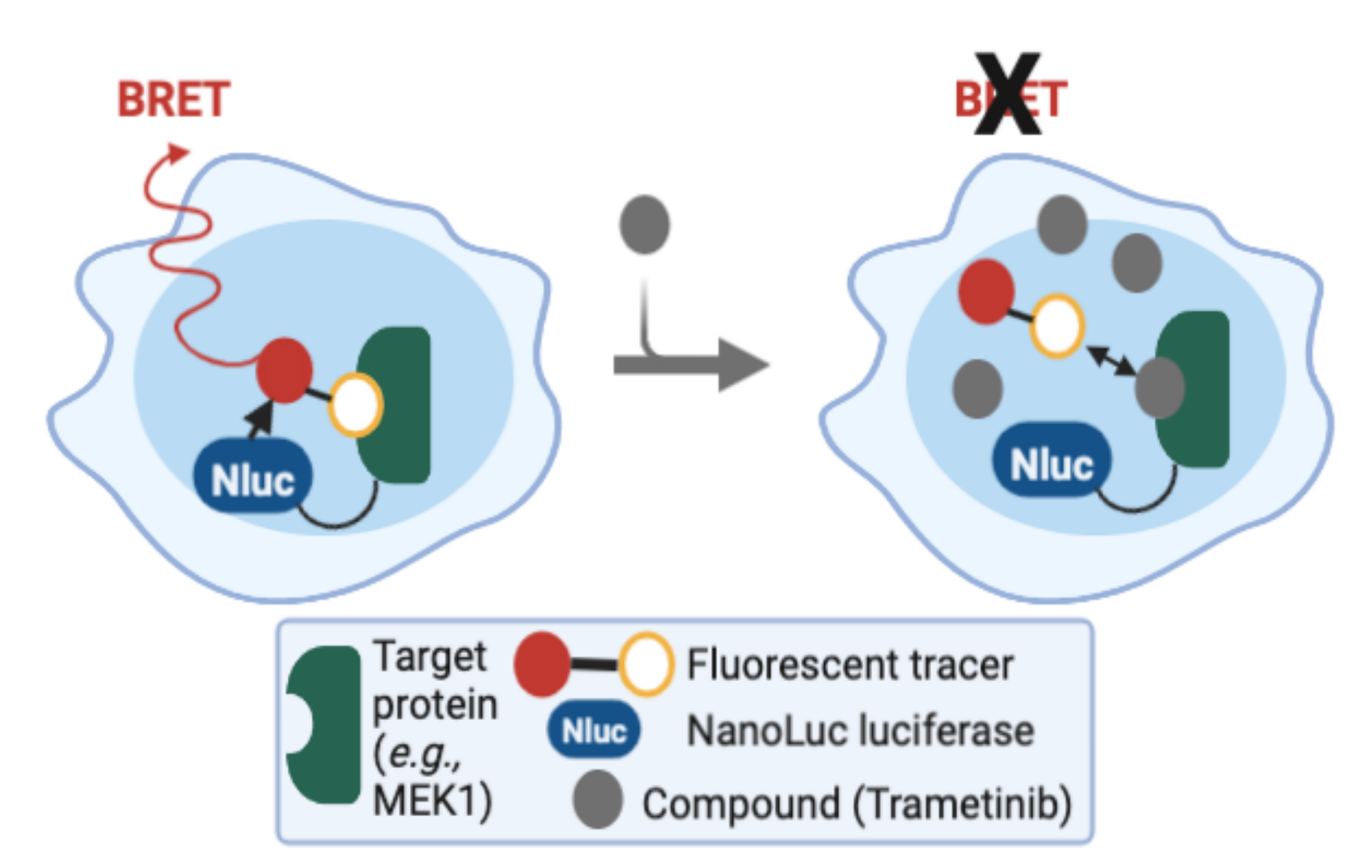
Overview of the NanoBRET system. The NanoBRET system is composed of a target protein (e.g., MEK1) tagged with Nanoluciferase. This target protein is the donor. BRET signal is achieved in the presence of a fluorescent tracer (the acceptor). This signal is competed away through the binding of unlabelled drug to the target protein.

NanoBRET is a modified form of the bioluminescence resonance energy transfer (BRET) assay for the measurement of protein–ligand interactions in living cells, for example, drug–target engagement (Figure 1) [6,7]. This proximity-based assay measures the interaction between a protein of interest (POI) and fluorescently labelled ligands. The POI is tagged with nanoluciferase (Nluc) that serves as an energy donor to the fluorescently labelled “tracer” energy acceptor, which binds constitutively to the POI. This interaction generates a BRET signal that is measured with a system that can measure luminescence and fluorescence. This instrumentation includes Promega’s Glomax plate reader and Olympus LV200 microscope. The BRET signal is lost in the presence of an unlabelled ligand, such as a drug that competitively binds to the POI, because of the displacement of the tracer.

A key advantage of the NanoBRET system over other technologies, such as FRET, is that it provides wider spectral separations between the emissions of the energy donor and acceptor [5]. This provides greater dynamic range and a brighter signal, ideal for the measurement of sensitive changes in protein–ligand binding and drug–target engagement. The protocol presented here describes the engineering of mouse colorectal cancer organoids for the measurement of drug–target engagement in a living 3D organoid setting.

## Materials and reagents

**Critical:** Follow manufacturer’s guidelines for the storage of materials and reagents. Aliquot reagents to reduce freeze–thaw cycles to once or twice.

**Biological materials**

1. HEK293T cell line [American Type Culture Collection (ATCC), catalog number: CRL-3216]

2. VAPK mouse organoids (*Villin-Cre^ERT2^: Apc^fl/fl^ Kras^G12D^/+ Trp53^fl/fl^*) are murine colorectal cancer organoids (gift from Sansom lab, CRUK SI) [3,8]

**Reagents**

1. Advanced Dulbecco’s modified Eagle medium/nutrient mixture F-12 (ADMEM/F12) (Gibco, catalog number: 12634-028)

2. GlutaMAX-I supplement (Gibco, catalog number: 35050-079)

3. HEPES (Gibco, catalog number: 15630-056)

4. Penicillin/streptomycin (Gibco, catalog number: 15140-122)

5. N2 supplement (Gibco, catalog number: 17502-048)

6. B27 supplement (Gibco, catalog number: 17504-044)

7. Mouse recombinant EGF (Invitrogen, catalog number: PMG8041)

8. Mouse recombinant Noggin (Peprotech, catalog number: 250-38)

9. TrypLE Express (Gibco, catalog number: 12604-021)

10. Opti-MEM^TM^ I reduced serum media, without phenol red (Gibco, catalog number: 11058021)

11. Dulbecco’s modified Eagle medium (DMEM) plus GlutaMAX-I (Gibco, catalog number: 31966-021)

12. Fetal bovine serum (FBS) (Gibco, catalog number: A5256801)

13. Blasticidin S HCl (Gibco, catalog number: A1113903)

14. Matrigel growth factor reduced (GFR) basement membrane matrix (Corning, catalog number: 356231)

15. Trypan Blue stain (0.4%) (Invitrogen, catalog number: T10282)

16. Trametinib-bodipy [Dar lab (MSKCC)]; synthesised as previously reported [9]

17. Trametinib (Selleckchem, catalog number: S2673)

18. Nano-Glo(R) fluorofurimazine in vivo substrate (Promega, catalog number: N4100)

19. Tracer dilution buffer (Promega, catalog number: N2191)

20. FuGENE HD transfection reagent (Promega, catalog number: E2311)

21. Polybrene infection/transfection reagent (Sigma-Aldrich, catalog number: TR-1003)

22. psPAX2 plasmid vector (Addgene, catalog number: 12260)

23. pCMV-VSV-G plasmid vector (Addgene, catalog number: 8454)

24. pLV-CMV-MEK1-Nluc-CMV-EGFP-Bsd (synthesised from VectorBuilder)

25. NanoBRET Nano-Glo(R) furimazine substrate (Promega, catalog number: N1571)

**Critical:** We obtained trametinib-bodipy directly from Arvin Dar (MSKCC). This reagent can be obtained from the Dar lab or synthesised using their published methods. We are in the process of synthesising a new batch from our collaborator at the University of Strathclyde.

**Solutions**

1. Growth medium for HEK293T cell line (see Recipes)

2. Basal organoid medium (see Recipes)

3. Organoid culture medium (see Recipes)

**Recipes**

**1. Growth medium for HEK293T cell line**

**Table:** 

Reagent	Stock concentration	Final concentration	Volume
DMEM/GlutaMAX-I	-	-	445 mL
FBS	100%	10%	50 mL
Penicillin/streptomycin	100×	1×	5 mL
Total	-	-	500 mL

*Note: Store at 4 °C for up to 1 month and prewarm to 37 °C using a water bath prior to usage.*

**2. Basal organoid medium**

**Table:** 

Reagent	Stock concentration	Final concentration	Volume
Advanced DMEM/F12	-	-	485 mL
GlutaMAX-I		1×	5 mL
HEPES		10 mM	5 mL
Penicillin/streptomycin		1×	5 mL
Total	-	-	500 mL

*Note: Store at 4 °C for up to 1 month and prewarm to 37 °C using a water bath prior to usage.*

**3. Organoid culture medium**

**Table:** 

Reagent	Stock concentration	Final concentration	Volume
Basal organoid medium	-	-	45 mL
N2 supplement	100×	1×	0.5 mL
B27 supplement	50×	1×	1 mL
EGF	1 μg/mL	50 ng/mL	2.5 mL
Noggin	5 μg/mL	100 ng/mL	1 mL
Total	-	-	50 mL

*Note: Store at 4 °C for up to 2 weeks and prewarm to 37 °C using a water bath prior to usage. Store supplements at -20 °C.*

**Laboratory supplies**

1. 6-well cell culture–treated plate (Thermo Fisher Scientific, catalog number: 140685)

2. T75 U-shaped cell culture flasks (Corning, catalog number: 15380591)

3. Nunc 96 solid white plate (Thermo Scientific, catalog number: 10346331)

4. 1.5 mL microtubes (Eppendorf, catalog number: 0030120086)

5. 15 mL centrifuge tube (Falcon, catalog number: 115074110)

6. 50 mL centrifuge tube (Falcon, catalog number: 11829650)

7. 2 mL aspirating pipette (Corning, catalog number: 9186)

8. 5 mL serological pipette (Corning, catalog number: 4050)

9. 10 mL serological pipette (Corning, catalog number: 4100)

10. 0.5–10 μL pipette tips (TipOne, catalog number: S1111-3810)

11. 20–200 μL pipette tips (TipOne, catalog number: S1113-1716)

12. 100–1,000 μL pipette tips (TipOne, catalog number: S1111-6811)

13. Cellometer SD100 slides (Revvity, catalog number: CHT4-SD100-002)

14. 40 μm sterile cell strainer (Greiner, catalog number: 542040)

## Equipment

1. Biological safety cabinet (Nuaire, catalog number: NU4374005)

2. Cell culture incubator (37 °C, 5% CO_2_) (Eppendorf, catalog number: 170R-230-1000)

3. Refrigerated centrifuge (Sigma, catalog number: 3-16KL)

4. Water bath (Thermo Fisher Scientific, catalog number: TSGP20)

5. Nexcelom automated cell counter system (Revvity, catalog number: CMT-A2K)

6. CoolCell freezer container (Corning, catalog number: CLS432002)

7. Inverted microscope (Zeiss, model: Axiovert A1)

8. Single-channel variable pipette, 0.1–2 μL (Gilson, catalog number: FA10001P)

9. Single-channel variable pipette, 2–20 μL (Gilson, catalog number: FA10003P)

10. Single-channel variable pipette, 20–200 μL (Gilson, catalog number: FA10005P)

11. Single-channel variable pipette, 100–1,000 μL (Gilson, catalog number: FA10006P)

12. Fridge (Biocold, catalog number: BIO155FRSSL)

13. -20 °C freezer (Biocold, catalog number: BIO102FZSS)

14. -80 °C freezer (Panasonic, catalog number: KMDU53Y1E)

15. Pipette controller (Falcon, catalog number: 357804)

16. Glomax Discover plate reader (Promega, catalog number: GM3000)

## Procedure

**A. Generation of organoid-compatible plasmid vector**

**A1. Design and synthesis of lentiviral nanoluciferase (Nluc) plasmid vector**

**Figure 2.**
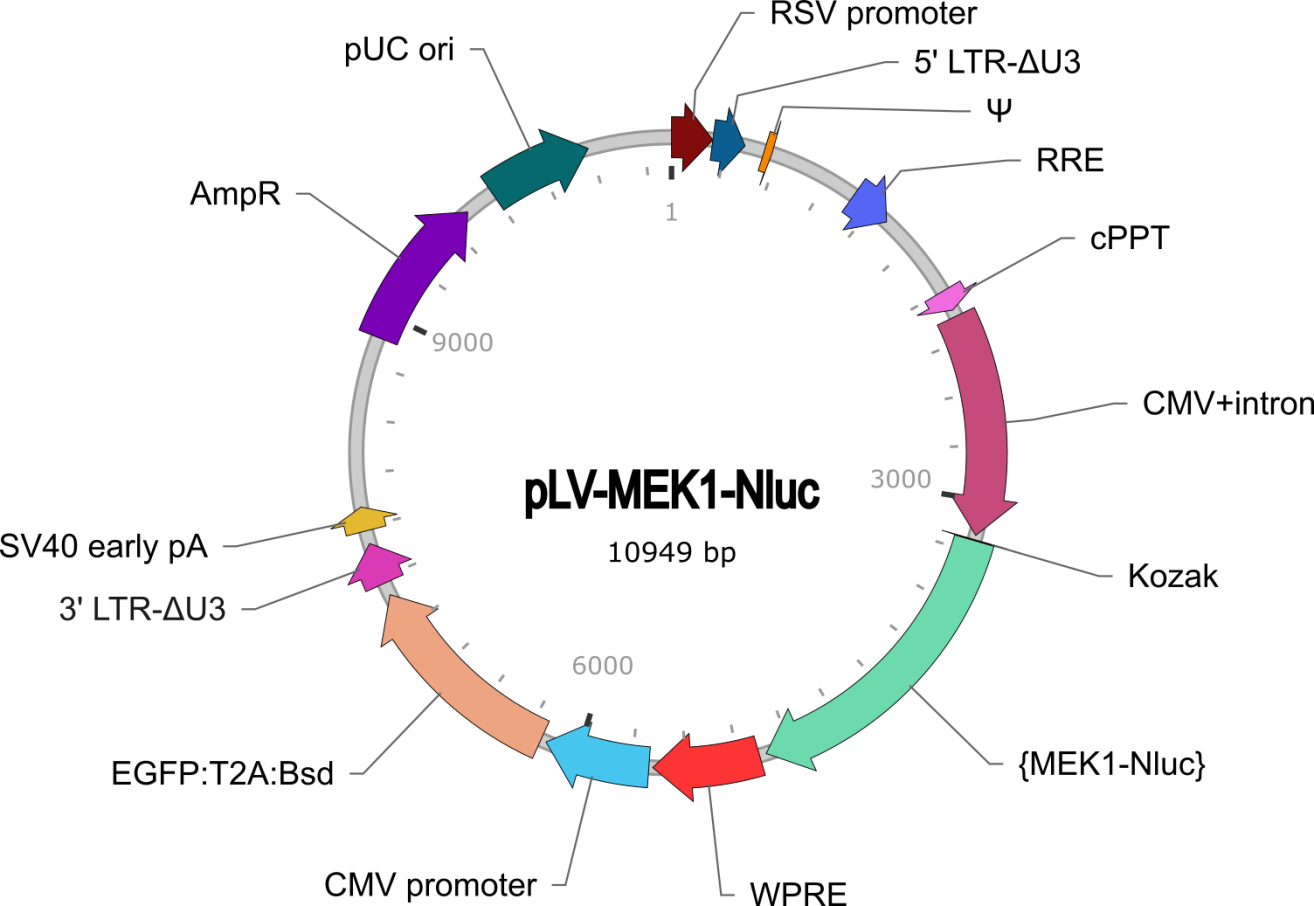
Vector map of pLV-CMV-MEK1-Nluc-CMV-EGFP-Bsd. The map includes MEK1-Nluc under the CMV promoter. Nluc organoids are GFP-positive and blasticidin-resistant. Plasmid DNA can be generated using ampicillin selection of bacterial expression system.

Using the DNA sequence of MEK1 from the NCBI database, we synthesised the *MEK1-Nluc* compatible with the organoid NanoBRET assay. Removing the stop codon, we then tagged the Nluc sequence to the C-terminal of MEK1 separated by a GTTT linker. The final sequence was synthesised and cloned into a second-generation lentiviral plasmid (Figure 2, File S1) by VectorBuilder.

**A2. Lentiviral production**

HEK293T (passage 3–10) can be used to manufacture viral particles through lentivirus plasmid packaging. We used second-generation lentiviral plasmids and packaging system due to the improved safety of lentivirus vector production and increased transfection efficiency.

**Day 1: Seed HEK293T cells**

**Critical:** Seed HEK293T cells into 75 cm^2^ U-shaped cell culture flasks with 10 × 10^6^ cells per flask for lentiviral plasmid transfection on day 2. A single 90% confluent 75 cm^2^ flask of HEK293T cells will provide enough cells for five 75 cm^2^ flasks.

1. Detach HEK293T cells from the flask (25 min)

a. Remove the culture medium using 2 mL aspirating pipettes.

b. Wash cells with 5 mL of PBS and aspirate.

c. Add 5 mL of TrypLE Express to cells.

d. Detach cells by incubating the flask at 37 °C for 5 min.

e. Generate a single-cell suspension by gentle trituration of cells.

f. Add 10 mL of culture medium to deactivate the activity of TrypLE Express.

g. Transfer contents from the flask into a 15 mL Falcon tube.

h. Centrifuge cells at 200× *g* for 5 min at room temperature.

i. Remove supernatant.

2. Count and seed cells (15 min)

a. Resuspend cells in 1 mL of culture medium.

b. Add 40 μL of cell suspension to a 1.5 mL Eppendorf tube. Add 40 μL of Trypan Blue and mix.

c. Pipette 20 μL of this sample into a Cellometer counting chamber and calculate the cell number using an automated cell counter.

d. Dilute cell suspension to 5 × 10^6^ cells/mL and plate 1 mL of cell suspension into two 75 cm^2^ flasks. Add 9 mL of Opti-MEM media supplemented with 1% FBS.

e. Incubate flasks at 37 °C in a 5% CO_2_ incubator overnight.

*Note: HEK293T cells attach and proliferate quickly; therefore, viral transfection can be performed the following day or after 30 h. The target confluency for transfection is 70% to prevent overconfluency during transfection and viral production steps.*

**Day 2: Transfect HEK293T cells**

In this step, the pLV-CMV-MEK1-Nluc-CMV-EGFP-Bsd lentiviral plasmid was used in combination with psPAX2 packaging plasmid and pCMV-VSV-G envelope plasmid.

3. Prepare transfection solution (30 min)

a. Defrost plasmid vectors. Use Fugene HD transfection reagent as the transfection carrier. Pre-label a 1.5 mL Eppendorf tube for the lentiviral plasmid vector.

b. To 452 μL of serum-free Opti-MEM media, add 36 μL of Fugene HD transfection reagent.

c. Pipette and add 5 μg of psPAX2, 5 μg of pCMV-VSV-G, and 2 μg of pLV-CMV-MEK1-Nluc-CMV-EGFP-Bsd to the Opti-MEM diluent.

d. Mix gently and allow the tube to sit for 15–20 min at room temperature.

4. HEK293T transfection (10 min)

a. After 20 min, the transfection complexes should form in the tube.

b. Retrieve the HEK293T cells from the previous day. The cells should have attached to the flask.

c. Gently transfer 250 μL of transfection complexes in a dropwise manner to each flask of HEK293T cells.

d. Incubate flasks at 37 °C in a 5% CO_2_ incubator for 12–18 h.

**Critical:** Pipette plasmids and transfection reagents gently and slowly. Avoid contact with the wall of the Eppendorf tubes. All transfected flasks should be handled very gently.

**Day 3: Media change 12–18 h after successful transfection**

5. Removal of transfection complexes (10 min)

a. From 12 to 18 h post-transfection, aspirate the media containing transfection complexes from the flasks.

b. Replace media with 5 mL of basal organoid media.

c. Incubate flasks at 37 °C in a 5% CO_2_ incubator overnight.

**Day 4: Production of lentiviral particles**

6. Lentiviral supernatant collection (30 min)

a. Pipette basal organoid media containing lentiviral particle into a pre-labelled 15 mL Falcon tube. Store this fraction at 4 °C.

b. Replace with 5 mL of fresh basal organoid media.

c. Incubate flasks at 37 °C in a 5% CO_2_ incubator for 8 h.

d. Collect another fraction of the lentiviral particle.

e. Combine fractions and centrifuge at 300× *g* for 5 min at 22 °C.

f. Lentiviral particle fractions can be concentrated by using polyethylene glycol (PEG) or commercial reagents. Measure viral titre using commercially available ELISA kits or fluorescence microscopy [10]. Based on our experiments, lentiviral titre value varies between 1 × 10^6^ and 1 × 10^7^ transducing units (TU)/mL.

g. Aliquot 1 mL of lentiviral particles supernatant or 50 μL of concentrated virus in 1.5 mL Eppendorf tubes.

h. Store aliquots at -80 °C.

**Pause point:** Concentrated virus should be used within 1 year.

**Caution:** Wear appropriate PPE during lentiviral production and transduction. Adhere to your local EPA guidelines for virus production and waste handling.

**B. Organoid culture**

**Day 5: Transduction of organoids**

In this step, mouse VAPK intestine tumour organoids expressing oncogenic Kras plus loss of Apc and p53 (*Villin-Cre^ERT2^: Apc^fl/fl^ Kras^G12D^/+ Trp53^fl/fl^*) are used for the viral infection experiment. Organoids were generated from mouse tissues following established protocols [11,12]. For this project, we used aliquots of frozen organoids.

1. Single-cell organoid suspension (40 min)

a. Harvest organoids into 15 mL Falcon tubes; use a maximum of six wells of a 6-well plate per tube.

b. Dissociate organoids by trituration using 200 μL pipette tips.

c. Centrifuge organoids at 200× *g* for 5 min at 4 °C. Aspirate the supernatant.

d. Add 5 mL of basal organoid media and triturate vigorously to separate the organoids from the Matrigel basement membrane matrix.

e. Centrifuge organoids at 200× *g* for 5 min at 4 °C. Aspirate the supernatant.

f. Resuspend organoids in 1 mL of TrypLE Express.

g. Incubate for 8 min at 37 °C in the water bath. Triturate every 3 min to create a single-cell suspension of organoids.

2. Organoid infection (30 min)

a. To the 1 mL of single cell suspension, add 9 mL of basal organoid media.

b. Pass the single-cell suspension through a 40 μm cell strainer.

c. Centrifuge cells at 200× *g* for 5 min at 4 °C. Aspirate the supernatant.

d. Resuspend cells in 0.5 mL of basal organoid media.

e. Add 40 μL of cell suspension to a 1.5 mL Eppendorf tube. Add 40 μL of Trypan Blue and mix.

f. Pipette 20 μL of this sample into a Cellometer^®^ counting chamber and calculate the cell number using an automated cell counter.

g. Dilute cell suspension to 1 × 10^6^ cells/mL.

h. Plate 0.1 mL of cell suspension into two wells of a 6-well plate.

i. Add 1 mL of lentiviral particle supernatant with a titre value of 1 × 10^6^ TU/mL to 1 × 10^5^ single-cell organoids per well. Then, add 8 μg/mL of polybrene to the wells.

j. Incubate plate at 37 °C in a 5% CO_2_ incubator overnight.

k. Keep empty 6-well plates in the incubator for use on day 6.

*Note: Dissociation of tissue and formation of organoids have been discussed in other protocols. Use TrypLE Express instead of trypsin and make sure organoids are properly dissociated for infection. We recommend using organoids that have been passaged no more than 10 times after generation from the original tissue.*

**Critical:** Organoids are quite sensitive to the viral particles: test various concentrations of particles for organoid infection. In our experience, a multiplicity of infection (MOI) of 10 is appropriate for organoid infection. Polybrene is very important for organoid infection; infection will fail in its absence.

**Day 6: Embed transduced organoids in Matrigel**

3. Collection of infected organoids (20 min)

a. Defrost aliquots of Matrigel on ice.

b. Retrieve the 6-well plate containing the infected organoid suspensions.

c. Pipette the contents of the two wells into separate 15 mL Falcon tubes.

d. Add basal organoid media to a total of 10 mL to the Falcon tubes.

e. Centrifuge cells at 200× *g* for 5 min at 4 °C. Aspirate the supernatant.

f. Wash cells with 10 mL of basal organoid media.

g. Centrifuge cells at 200× *g* for 5 min at 4 °C. Aspirate the supernatant.

4. Plate organoids (40 min)

**Critical:** Avoid bubble formation when working with Matrigel.

a. Add 120 μL of Matrigel to the cells in each of the Falcon tubes.

b. Create six domes of 20 μL cell–Matrigel suspension in each well of the prewarmed 6-well plate.

c. Transfer plate to the 37 °C incubator for 25 min. This incubation will allow the Matrigel matrix to solidify, creating the 3D organoid structure.

d. After 25 min, add 2 mL of organoid culture medium to each well of the 6-well plate to the side of the well without disturbing the domes.

e. Incubate the plate at 37 °C in a 5% CO_2_ incubator for 3 days.

*Note: Prewarm the 6-well plates in the incubator before use.*

**Day 9: Organoid passage and selection of Nluc-positive organoids**

**Critical:** Antibiotic selection can be started after 3–4 days of organoid infection. In our experience, selection after 1–2 days will lead to toxicity and lack of organoid growth.

5. Single-cell organoid suspension (40 min)

a. Harvest organoids into 15 mL Falcon tubes; use a maximum of six wells of a 6-well plate per tube.

b. Dissociate organoids by trituration using 200 μL pipette tips.

c. Centrifuge cells at 200× *g* for 5 min at 4 °C. Aspirate the supernatant.

d. Add 5 mL of basal organoid media and triturate vigorously to separate the organoids from the Matrigel basement membrane matrix.

e. Centrifuge organoids at 200× *g* for 5 min at 4 °C. Aspirate the supernatant.

f. Resuspend organoids in 1 mL of TrypLE Express.

g. Incubate for 8 min at 37 °C in the water bath. Triturate every 3 min to create a single-cell suspension of organoids.

6. Organoid selection (40 min)

a. To the 1 mL of single-cell suspension, add 9 mL of basal organoid media.

b. Centrifuge cells at 200× *g* for 5 min at 4 °C. Aspirate the supernatant.

c. Add 240 μL of Matrigel to the cells in the Falcon tube(s).

d. Create six domes of 20 μL cell–Matrigel suspension in each well of the prewarmed 6-well plate.

e. Transfer the plate to the 37 °C incubator for 25 min.

f. After 25 min, add 2 mL of organoid culture medium to each well of the 6-well plate. Supplement the media with 5 μg/mL of blasticidin for the selection of Nluc-positive organoids.

g. Incubate the plate at 37 °C in a 5% CO_2_ incubator for 48 h.

**Critical:** Organoids are sensitive to antibiotic selection; therefore, determine the optimal antibiotic concentration using a dose-response experiment. The range of blasticidin concentration was 0–500 μg/mL for the dose-response curve. In this protocol, we used a plasmid that has blasticidin resistance and GFP tags. FACS experiment can also be used to purify Nluc-positive organoids after 48 h of antibiotic treatment.

**Day 11: Media change**

7. Inspect Nluc-positive organoids and perform media change (10 min)

a. Retrieve the plate and aspirate media.

b. Replace with 2 mL of fresh organoid culture media supplemented with 5 μg/mL of blasticidin.

c. Incubate the plate at 37 °C in a 5% CO_2_ incubator for 48 h.

**Critical note:** Due to the sensitivity of organoids to antibiotic selection, Nluc-negative organoids should undergo cell death after 48 h of incubation.

**Day 13: Organoid passage**

**Figure 3.**
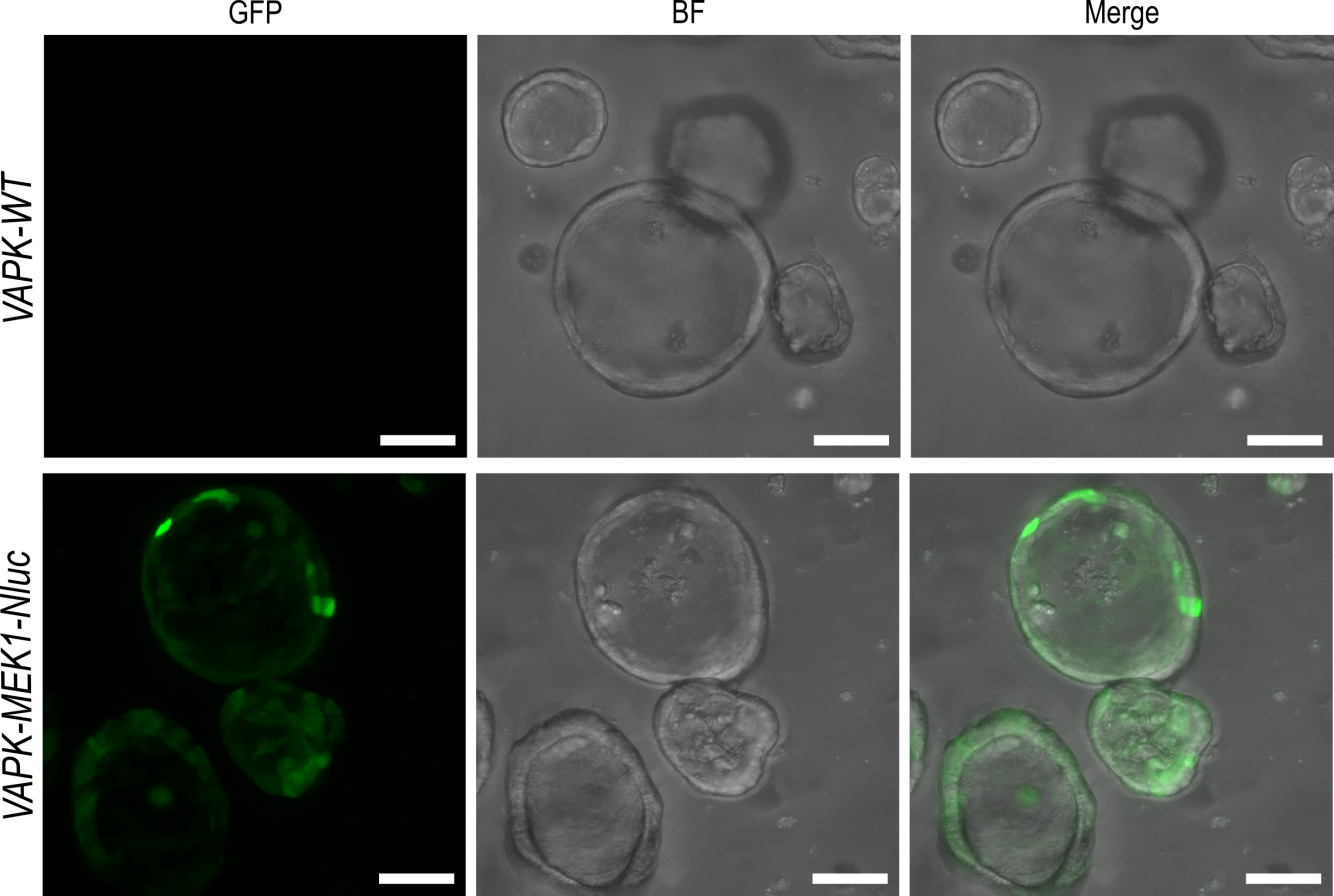
Selection of transduced organoids. Representative green fluorescent protein (GFP) and brightfield (BF) images of organoids negative for MEK1-Nluc (VAPK-WT) and organoids positive for MEK1-Nluc (VAPK-MEK1-Nluc). Scale bars, 50 μm.

Repeat the procedure on day 9 and split one well of organoids into three wells of a 6-well plate. Media should be added in the presence of blasticidin to ablate Nluc-negative organoids. Allow organoids to grow for 3 days. Transduced organoids are GFP-positive, as shown in Figure 3.

**Day 16: Validation of Nluc expression in organoids and organoid passage**

8. Seeding organoids for measuring Nluc expression (60 min)

a. Harvest cells from Nluc-positive organoids. As a control, harvest organoids that have not been infected with the Nluc reporter.

b. Dissociate organoids by trituration.

c. Centrifuge cells at 200× *g* for 5 min at 4 °C. Aspirate the supernatant.

d. Add 5 mL of basal organoid media and triturate vigorously to separate the organoids from the Matrigel basement membrane matrix.

e. Centrifuge organoids at 200× *g* for 5 min at 4 °C. Aspirate the supernatant.

f. Add 1 mL of TrypLE Express to the organoids.

g. Incubate for 8 min at 37 °C in the water bath.

h. Pipette up and down every 3 min to create a single-cell suspension of organoids.

i. To the 1 mL of single cell suspension, add 9 mL of basal organoid media.

j. Pass the single-cell suspension through a 40 μm cell strainer.

k. Centrifuge cells at 200× *g* for 5 min at 4 °C. Aspirate the supernatant.

l. Resuspend cells in 0.5 mL of basal organoid media.

m. Add 40 μL of cell suspension to a 1.5 mL Eppendorf tube. Add 40 μL of Trypan Blue and mix.

n. Pipette 20 μL of this sample into a Cellometer^®^ counting chamber and calculate the cell number using an automated cell counter.

o. Dilute the cell suspension to 1 × 10^5 ^cells per mL of Matrigel.

p. Plate 20 μL of cell suspension into at least three wells of a white opaque-bottom 96-well plate.

q. After 25 min, add 0.1 mL of organoid culture medium to each well of the 96-well plate.

r. Incubate the plate at 37 °C in a 5% CO_2_ incubator for 48 h.

**Day 18: Measurement of Nluc expression in organoids**

**Figure 4.**
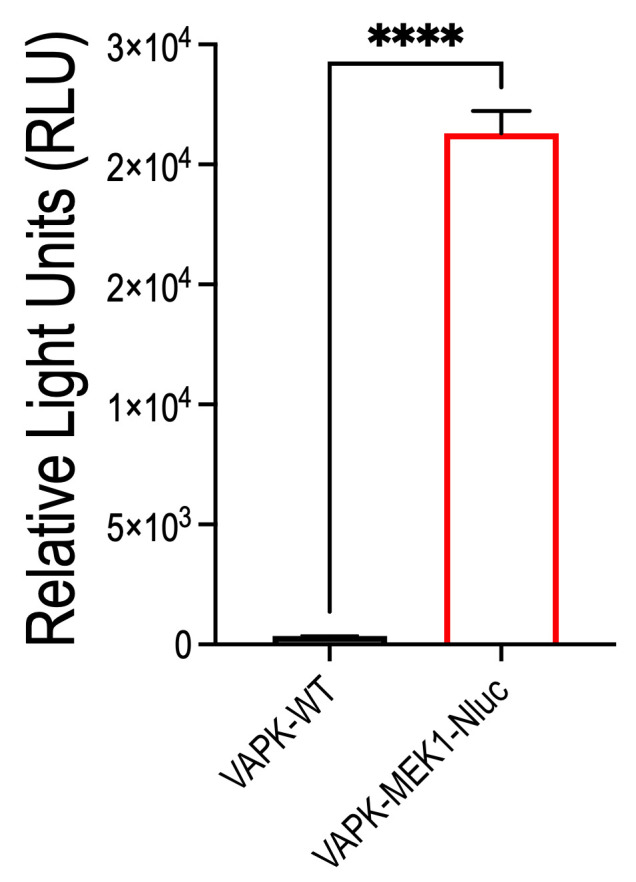
Expression of MEK1-Nluc. Quantification of MEK1-Nluc bioluminescence in mouse VAPK intestine tumour organoids (*Villin-Cre^ERT2^: Apc^fl/fl^ Kras^G12D^/+ Trp53^fl/fl^*). The bar graph represents the mean data obtained from triplicate wells from a representative experiment ± SEM. ****P < 0.0001 by Student’s t-test.

9. Glomax plate reader to measure Nluc expression (15 min)

a. Retrieve the 96-well plate. Allow to cool at room temperature for 3 min.

b. Prepare a 10 mM stock of Promega Nano-Glo fluorofurimazine in vivo substrate. Dilute this stock to 1:250 with Opti-MEM.

c. Pipette 50 μL of this dilution to each well of the 96-well plate. Incubate for 2 min.

d. Measure Nluc expression using any luminometer plate reader. We used the Glomax Discover plate reader with the Nano-Glo protocol; our results are shown in Figure 4.

**Day 19: Passaging and storing cells**

Once expression of Nluc has been confirmed, passage cells to continue NanoBRET experiments and freeze aliquots for future experiments and sharing.

10. Plate organoids for NanoBRET experiment (60 min)

a. Harvest cells from Nluc-positive organoids. As a control, harvest organoids uninfected with the Nluc reporter.

b. Dissociate organoids by trituration.

c. Centrifuge cells at 200× *g* for 5 min at 4 °C. Aspirate the supernatant.

d. Add 5 mL of basal organoid media and triturate vigorously to separate the organoids from the Matrigel basement membrane matrix.

e. Centrifuge organoids at 200× *g* for 5 min at 4 °C. Aspirate the supernatant.

f. Add 1 mL of TrypLE Express to organoids.

g. Incubate for 8 min at 37 °C in the water bath.

h. Pipette up and down every 3 min to create a single-cell suspension of organoids.

i. To the 1 mL of single-cell suspension, add 9 mL of basal organoid media.

j. Pass the single-cell suspension through a 40 μm cell strainer.

k. Centrifuge cells at 200× *g* for 5 min at 4 °C. Aspirate the supernatant.

l. Resuspend cells in 0.5 mL of basal organoid media.

m. Add 40 μL of cell suspension to a 1.5 mL Eppendorf tube. Add 40 μL of Trypan Blue and mix.

n. Pipette 20 μL of this sample into the Cellometer^®^ counting chamber and calculate the cell number using an automated cell counter.

o. Dilute the cell suspension to 2 × 10^5^ cells per mL of Matrigel.

p. Plate 20 μL of organoid suspension into at least triplicate wells of a white opaque-bottom 96-well plate. Be sure to prepare sufficient wells for all experimental conditions.

q. After 25 min, add 0.1 mL of organoid culture medium to each well of the 96-well plate.

r. Incubate the plate at 37 °C in a 5% CO_2_ incubator for 48 h.

*Note: We recommend preparing at least 10% extra volume of organoid suspension to account for the volume lost during the pipetting of viscous Matrigel.*

**C. NanoBRET target engagement**

**Day 21: NanoBRET competitive assay**

*MEK1-Nluc* expression was confirmed as shown above; here, we measure POI–drug binding. We tested the impact of treating organoids that express *MEK1-Nluc* with (i) the potent MEK binder/inhibitor trametinib, (ii) the multi-kinase inhibitor regorafenib, and (iii) both compounds together as a combination. DMSO was used as the vehicle, and the binding assay was done with 10, 100, and 1,000 nM of trametinib, 100 nM of regorafenib, and 100 nM of each compounds for combination treatment. Trametinib-bodipy was used as the tracer at 0.25 μM.

1. NanoBRET experiment on MEK1-Nluc organoids (40 min)

a. Preparation and addition of test compounds

i. Prepare a stock of every dose of trametinib and regorafenib at 1,000× final concentration in 100% DMSO (for example, 10 μM stock for the 10 nM dose of trametinib or 100 μM stock for the 100 nM dose of regorafenib).

ii. Dilute these stocks or DMSO to a 10× final concentration in Opti-MEM.

iii. Add 10 μL per well of the diluted DMSO, trametinib, and regorafenib to the *MEK1-Nluc* organoids in the 96-well plate. Doses should be added in triplicate.

iv. Mix on an orbital shaker for 20 s at 50 rpm.

b. Preparation of trametinib-bodipy

i. Prepare a 100× solution of trametinib-bodipy in 100% DMSO. Here, we used a 0.25 μM dose of trametinib-bodipy; therefore, we prepared a 25 μM stock solution.

ii. Dilute trametinib-bodipy to 20× final concentration in tracer dilution buffer.

iii. Add 5 μL of the 20× trametinib-bodipy to the *MEK1-Nluc* organoids containing DMSO, trametinib, and regorafenib.

iv. Shake the 96-well plate on an orbital shaker for 20 s.

v. Incubate plate at 37 °C with 5% CO_2_ for 2 h.

**Critical:** During this incubation period, target engagement and competitive binding assay will occur. Varying time can be used for other compounds, depending on the kinetics of the compound and the protein of interest.

**Figure 5.**
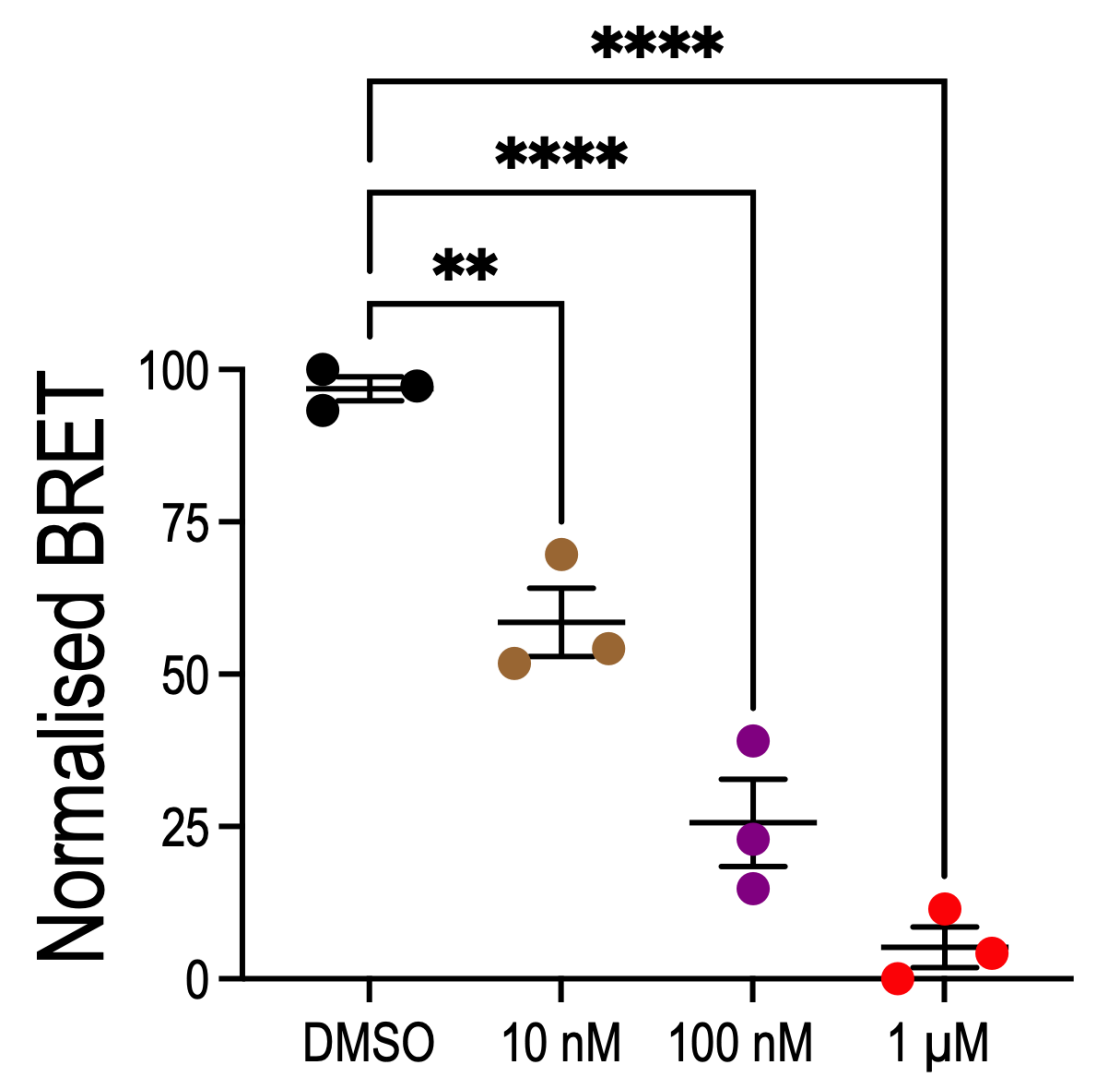
Engagement of trametinib with MEK1-Nluc. NanoBRET assay showing the binding of trametinib to MEK1 in a dose-dependent manner in organoids. DMSO is the trametinib-bodipy tracer alone at a 0.25 μM dose. The 10 nM, 100 nM, and 1 μM are varying concentrations of trametinib in the presence of 0.25 μM trametinib-bodipy. Successful drug displacement occurs when the trametinib-treated condition is significantly different from DMSO. Plot shows mean ± S.E.M. from n = 3 biological replicates. ****P < 0.0001, **P < 0.005, one-way ANOVA with Tukey correction.

**Figure 6.**
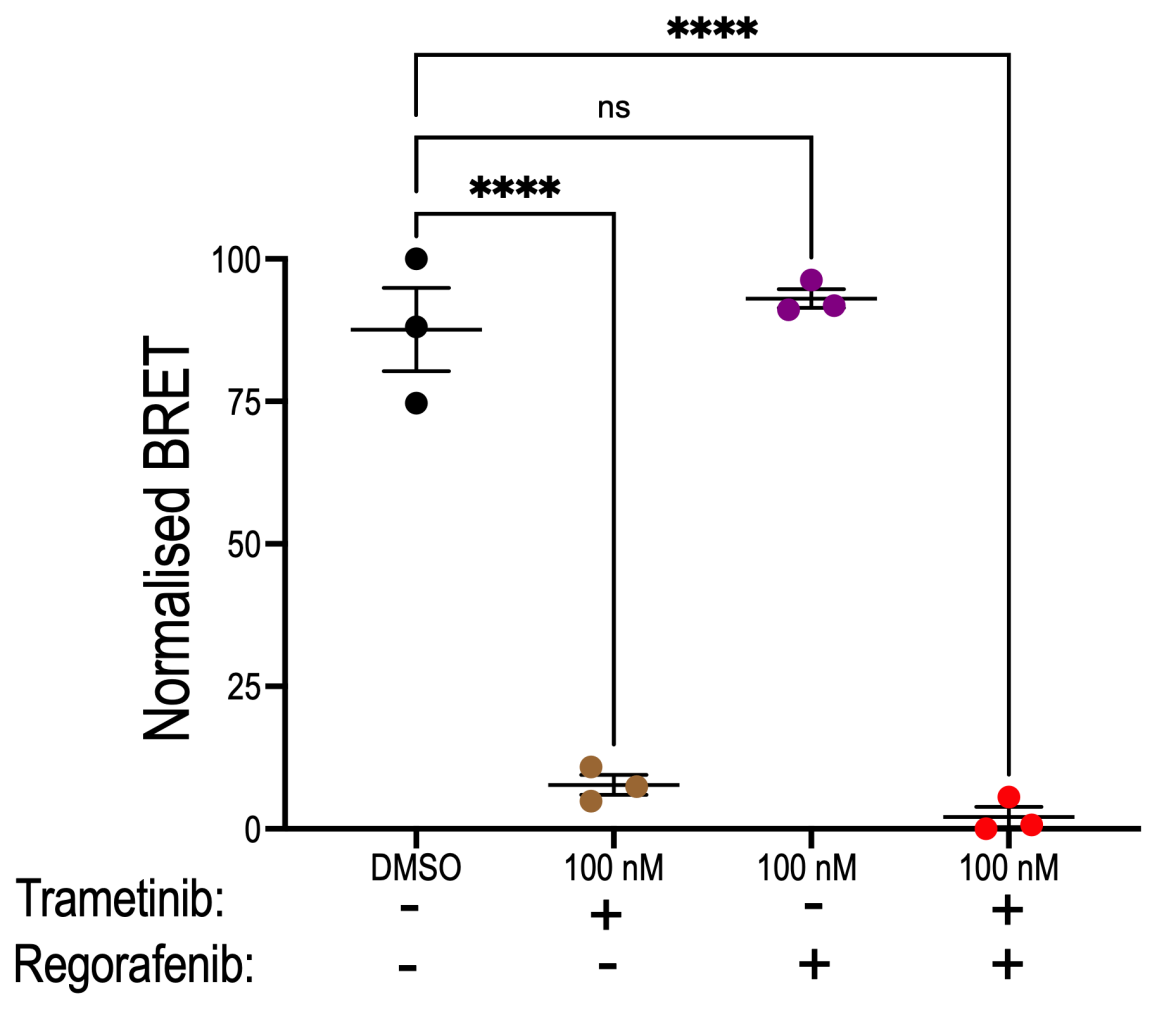
Impact of regorafenib on the engagement of trametinib with MEK1-Nluc. NanoBRET assay showing the binding of trametinib to MEK1 in the presence of regorafenib in organoids. Regorafenib failed to bind to MEK1 as expected and did not affect the binding of trametinib to MEK1. Plot shows mean ± S.E.M. from n = 3 biological replicates. ****P < 0.0001, one-way ANOVA with Tukey correction.

2. NanoBRET measurement (15 min)

a. Retrieve the 96-well plate. Allow to cool at room temperature for 3 min.

b. Prepare a 10 mM stock of Promega Nano-Glo fluorofurimazine in vivo substrate. Dilute this stock to 1:250 with Opti-MEM.

c. Pipette 50 μL of this dilution to wells containing the treated *MEK1-Nluc* organoids.

d. Incubate for 3 min at room temperature.

e. Shake the 96-well plate on an orbital shaker for 20 s.

f. Measure NanoBRET using any luminometer plate reader. Here, we used the Glomax discover plate reader with the standard NanoBRET protocol: we measured donor emission at 495 nm and acceptor emission at 600 nm, with integration time 0.3 s. The protocol calculates the BRET value as a ratio of the acceptor emission value and the donor emission value; our normalised result is shown below (Figures 5 and 6).

*Note: For optimal BRET signal, measure NanoBRET within 40 min of substrate addition.*

## Data analysis

NanoBRET signal was calculated as a ratio of the acceptor signal to donor signal. This ratio was plotted as the normalised percentage to the DMSO control. Graphs represent the mean ± S.E.M. of three independent biological replicates with three technical replicates each. No data were excluded. Statistical significance between two groups was determined by an unpaired Student’s t-test, and comparisons among more than two groups were done by one-way ANOVA with Tukey post hoc correction. All statistical tests and graphing were done in GraphPad Prism software (version 11.0), and differences were considered significant at P < 0.05.

## Validation of protocol

This NanoBRET-based protocol provides the first detailed roadmap for measuring drug–target engagement in 3D organoids. We developed and validated this protocol in mouse colorectal cancer organoids. This protocol should be adaptable to other organoid systems with the optimisation of various critical steps. The most challenging parts of this protocol are designing the organoid-compatible plasmid vector, getting the *MEK1-Nluc* into organoids, and expressing the luciferase enzyme for substrate catalysis. When performed using the protocol described, the relative emission by the Nluc organoids will be 20,000 RLU higher than by base organoids. This high NanoBRET emission provides a large window for quantifying POI–drug binding in vivo. We have used this protocol to measure the engagement of trametinib to MEK1 in a dose-dependent manner (Figure 5). Additionally, we showed that regorafenib failed to engage MEK1, and the combination with trametinib did not impact the engagement of trametinib to MEK1 (Figure 6).

## General notes and troubleshooting

**General notes**

The NanoBRET assay is an increasingly popular tool for measuring drug–target engagement in live cells; we have adapted this for colorectal cancer organoids. While the basic principle of the protocol should be useful for other types of organoids, some optimisation will be needed. For example, drug–target engagement may be done in the absence of the 3D matrix for optimal signal.

**Troubleshooting**

**Problem 1:** Failure to generate Nluc organoids (related to steps B1–4).

Potential causes: A lack of Nluc-positive organoids can be caused by several factors, including the use of the wrong plasmid vector, poor organoid proliferation, poor lentiviral particle titre, and toxicity from viral particles.

Solutions: Manufacture lentiviral plasmids that are compatible for organoid transduction. Increase organoid number compared to the viral titre, if organoids are slow-growing. Repeat the lentiviral transfection step with multiple flasks to increase lentiviral titre and concentrate virus. Viral particles can be toxic to some organoids, so incubate organoids for a short period of time compared to the overnight incubation used in this protocol. We found that the transfection efficiency is 85%, and our selection approaches with blasticidin and GFP allowed us to attain 100% Nluc-positive organoids.

**Problem 2:** Lack of signal from Nluc organoids (related to steps B8–9).

**Figure 7.**
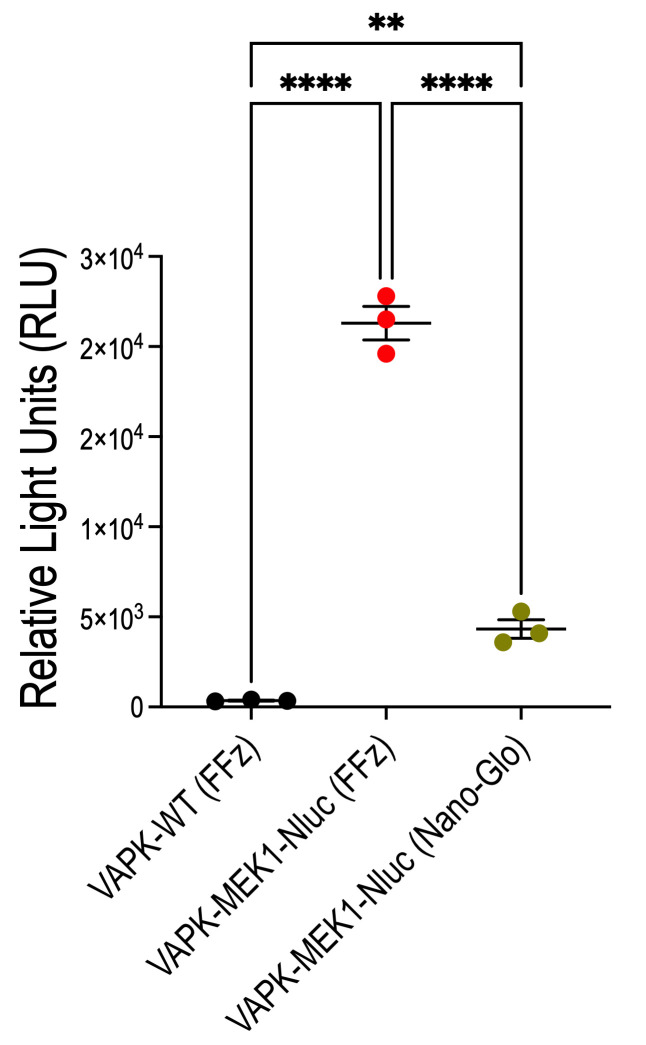
Comparison of fluorofurimazine (FFz) substrate with furimazine substrate (Nano-Glo). FFz shows improved readout for the expression of MEK1-Nluc compared to the Nano-Glo. Plot shows mean ± S.E.M. from n = 3 biological replicates. ****P < 0.0001, **P < 0.005, one-way ANOVA with Tukey correction.

Potential solution: The Nano-Glo substrate (furimazine) from Promega was optimised for the measurement of Nluc expression in 2D cell lines. During the development of this protocol, the same substrate gave a relatively low signal. Interestingly, Promega then developed the fluorofurimazine substrate that works well with 3D organoids and gave significantly improved signals in Nluc-positive organoids (Figure 7).

## Supplementary information

The following supporting information can be downloaded here:

1. File S1. pLV-CMV-MEK1-Nluc-CMV-EGFP-Bsd
